# Close Link Between Harmful Cyanobacterial Dominance and Associated Bacterioplankton in a Tropical Eutrophic Reservoir

**DOI:** 10.3389/fmicb.2018.00424

**Published:** 2018-03-12

**Authors:** Iame A. Guedes, Caio T. C. C. Rachid, Luciana M. Rangel, Lúcia H. S. Silva, Paulo M. Bisch, Sandra M. F. O. Azevedo, Ana B. F. Pacheco

**Affiliations:** ^1^Instituto de Biofísica Carlos Chagas Filho, Universidade Federal do Rio de Janeiro, Rio de Janeiro, Brazil; ^2^Instituto de Microbiologia Professor Paulo de Góes, Universidade Federal do Rio de Janeiro, Rio de Janeiro, Brazil; ^3^Departamento de Botânica, Museu Nacional, Universidade Federal do Rio de Janeiro, Rio de Janeiro, Brazil

**Keywords:** *16S rDNA*, *Microcystis*, *Synechococcus*, *Cylindrospermopsis*, cyanobacterial bloom, microbial community, Illumina

## Abstract

Cyanobacteria tend to become the dominant phytoplankton component in eutrophic freshwater environments during warmer seasons. However, general observations of cyanobacterial adaptive advantages in these circumstances are insufficient to explain the prevalence of one species over another in a bloom period, which may be related to particular strategies and interactions with other components of the plankton community. In this study, we present an integrative view of a mixed cyanobacterial bloom occurring during a warm, rainy period in a tropical hydropower reservoir. We used high-throughput sequencing to follow temporal shifts in the dominance of cyanobacterial genera and shifts in the associated heterotrophic bacteria community. The bloom occurred during late spring-summer and included two distinct periods. The first period corresponded to *Microcystis aeruginosa* complex (MAC) dominance with a contribution from *Dolichospermum circinale*; this pattern coincided with high water retention time and low transparency. The second period corresponded to *Cylindrospermopsis raciborskii* and *Synechococcus* spp. dominance, and the reservoir presented lower water retention time and higher water transparency. The major bacterial phyla were primarily Cyanobacteria and Proteobacteria, followed by Actinobacteria, Bacteroidetes, Verrucomicrobia, and Planctomycetes. Temporal shifts in the dominance of cyanobacterial genera were not only associated with physical features of the water but also with shifts in the associated heterotrophic bacteria. The MAC bloom was associated with a high abundance of Bacteroidetes, particularly Cytophagales. In the second bloom period, Planctomycetes increased in relative abundance, five Planctomycetes OTUs were positively correlated with *Synechococcus* or *C. raciborskii* OTUs. Our results suggest specific interactions of the main cyanobacterial genera with certain groups of the heterotrophic bacterial community. Thus, considering biotic interactions may lead to a better understanding of the shifts in cyanobacterial dominance.

## Introduction

Cyanobacterial blooms occur in freshwater environments around the world, mainly as a result of eutrophication (Rigosi et al., 2014). These events cause deleterious environmental and socioeconomic effects, impacting the ecosystem due to disruption of the food webs and thus potentially decreasing biodiversity. Cyanobacterial blooms also impair the use of water by human populations (Paerl and Paul, 2012). During the last few decades, an expansion of cyanobacterial blooms has been recorded, and global warming is expected to further exacerbate this situation (Paerl, 2008; Visser et al., 2016).

Potentially harmful cyanobacteria tend to become the dominant phytoplankton component in eutrophic freshwater environments, especially during warmer seasons (Paerl and Huisman, 2008). Indeed, the main reported abiotic drivers of cyanobacterial blooms are increased nutrient loading (nitrogen and phosphorus) and rising temperatures (Kosten and Huszar, 2012; Lürling et al., 2017). Some bloom-forming cyanobacteria have efficient nutrient uptake and storage abilities and can use both inorganic and organic N and P pools (O'Neil et al., 2012; Paerl and Paul, 2012; Harke and Gobler, 2013). Generally, cyanobacteria will grow faster at higher temperatures compared to other phytoplankton groups, although growth rates can be differentially affected depending on the species considered (Paerl, 2008; Paerl and Paul, 2012; Lürling et al., 2013). Rising temperatures also intensify vertical stratification, and in this situation some bloom-forming cyanobacteria can be benefited due to their buoyancy ability (Kruk et al., 2010). Cells can accumulate at the surface and shade underlying layers, thus outcompeting other phytoplankton groups through light limitation (Reynolds, 2006; Harke et al., 2016). Additionally, buoyant cyanobacteria can access nutrients from deeper waters when epilimnion concentrations are diminished (Graham et al., 2016). These general observations, however, are insufficient to explain the prevalence of one species over another in a bloom period, which may be related to particular adaptive strategies and interactions with other components of the plankton community (Woodhouse et al., 2016; Wood et al., 2017).

In contrast to abiotic factors, the role of biotic interactions on cyanobacterial bloom dynamics has been less explored. Diverse biotic factors such as grazing, predation, parasitism and mutualism influence cyanobacterial biomass through interactions with other plankton components such as protozoans, zooplankton, bacteria, and viruses (Paerl and Otten, 2013; Gerphagnon et al., 2015; Ger et al., 2016; Steffen et al., 2017). Interactions between cyanobacteria and heterotrophic bacteria can be positive due to the exchange of nutrients and oxygen, which can benefit both microorganisms (Bagatini et al., 2014; Gerphagnon et al., 2015). Interactions may also be negative, as for cyanolytic bacteria (Van Wichelen et al., 2016; Osman et al., 2017). During cyanobacterial blooms, bacteria can be found directly attached to cyanobacterial cells or adjacent to them, occupying the cyanobacterial phycosphere (Louati et al., 2015). In many cases, these associations were reported under laboratory conditions, but several recent studies in natural environments have provided evidence of the close relation between cyanobacterial biomass and associated heterotrophic bacteria (Eiler and Bertilsson, 2004; Wu et al., 2007; Kormas et al., 2010; Cheng et al., 2011; Dziallas and Grossart, 2011; Li et al., 2011, 2015; Wilhelm et al., 2011; Steffen et al., 2012; Cai et al., 2013; Parveen et al., 2013; Louati et al., 2015; Woodhouse et al., 2016; Parulekar et al., 2017; Salmaso et al., 2017). Moreover, specific associations between some cyanobacterial genera and heterotrophic bacteria have recently been reported (Bagatini et al., 2014; Louati et al., 2015), pointing to a possible connection of those bacteria attached to cyanobacteria and their participation in cyanobacterial bloom dynamics. Some authors extend this view and suggest that microbial communities of distinct taxonomic composition can play similar functional roles in bloom events (Steffen et al., 2012). Clearly, the complexity of these microbial interactions is still little explored, and their impact in the ecophysiology of cyanobacteria is underestimated.

High-throughput sequencing has revolutionized microbial ecology in recent years, particularly through the characterization of metagenomes. By following temporal variation in the composition of a community, it is possible to reveal correlations and to infer interactions among taxa (Stubbendieck et al., 2016). This application can be explored to better understand cyanobacterial species dynamics during a bloom, and the accompanying bacterial community can be seen as an integral and essential part of these events.

In this study, we present an integrative view of a mixed cyanobacterial bloom occurring during a warm, rainy period in a tropical hydropower reservoir; we used high-throughput sequencing to follow temporal shifts in the dominance of cyanobacterial genera and the associated heterotrophic bacterial community. We also looked for correlations between temporal shifts in the bacterial community and abiotic factors.

## Materials and methods

### Sampling

Water samples were collected in the Funil Reservoir (22°30′S, 44°45′W), Rio de Janeiro, Brazil (Supplementary Figure 1), from October 2013 to March 2014. This is a eutrophic reservoir with an area of 40 km^2^, a volume of 8.9 × 10^6^ m^3^, maximum and medium depth of 70 and 22 m, respectively, and average residence time of 41.5 days (Soares et al., 2009; Rangel et al., 2012). The region in which the reservoir is located typically presents warm, rainy conditions during summer and cold, dry conditions during winter. The sampling period encompassed the end of spring and the summer.

Samples were collected from two locations: one in the central part of the reservoir (point 1) and the other near the dam (point 2). From one to three samples per month were obtained from the integrated euphotic zone (determined as 2.7 times the Secchi disk depth; Cole, 1994) for a total of 22 samples. Water temperature and pH were measured using a Yellow Spring multiparametric probe (model 600 QS), and the water transparency was determined using a Secchi disk. Water temperature and pH values were measured at 0.5-m intervals to the end of the euphotic zone, and average values are presented. Volumes of 0.3–1 L of water (depending on the phytoplankton density) from the integrated euphotic zone were filtered (Whatman GF/F, 0.7 μm) to collect cells and then stored at −20°C for DNA extraction.

### Nutrient analysis

Aliquots of water for total and dissolved nutrient analysis were collected in polypropylene tubes and stored at −20°C. For dissolved nutrient analysis, aliquots were filtered (Whatman GF/F, 0.7 μm) before storage. The soluble reactive phosphorus (SRP), ammonium, nitrate, nitrite, and total phosphorus (TP) were measured using flow injection analysis (FIAlab 2500) according to the manufacturer's instructions (FIALab Instruments Inc., Seattle, Washington).

### Quantitative analysis of phytoplankton

Aliquots of the integrated water samples were stored in amber glass vials with Lugol's solution. Phytoplankton abundance was determined by the Utermöhl method (Utermöhl, 1958) using an inverted optical microscope (Olympus BX-51). The biovolume (mm^3^ L^−1^) was estimated by multiplying the density of each species by the average volume of its cells (Hillebrand et al., 1999).

### DNA extraction and *16SrDNA* amplification

DNA was extracted from cells collected in filters (Whatman GF/F, 0.7 μm) using the Power Soil DNA Isolation Kit (Mo Bio) according to the manufacturer's instructions. DNA samples were quantified using a fluorimeter (Qubit, Thermo Fisher Scientific). Amplification of the v3-v4 region of *16S rDNA* genes was performed with the primers S-D-Bact-0341-b-S-17F (5′-CCTACGGGNGGCWGCAG-3′) and S-D-Bact-0785-a-A-21R (5′-GACTACHVGGGTATCTAATCC-3′) (Klindworth et al., 2013) containing the appropriate adaptors for sequencing in the Illumina platform. Amplifications were performed in a 25 μl reaction mixture containing 12.5 μL of HiFi HotStart ReadyMix (KAPA Biosystems), 0.2 μM of each primer and 12.5 μg of DNA. The PCR program included an initial denaturation at 95°C for 3 min followed by 25 cycles of amplification (95°C for 30 s, 55°C for 30 s, and 72°C for 30 s) and a final step of 72°C for 5 min. Products were purified using magnetic beads (Agencourt AMPure XP, Beckman Coulter) and subjected to a second PCR to incorporate dual indices, as described in the 16S Metagenomic Sequencing Library Preparation Protocol for the Illumina MiSeq System. The size and quality of DNA in the final libraries were verified on a Bioanalyzer 2100 (Agilent) using a Bioanalyzer DNA 1000 chip (Agilent Technologies). After quantitative PCR with a KAPA Library Quantification Kit for Illumina (KAPA Biosystems), samples were normalized and pooled for sequencing.

### DNA sequencing and data analysis

Sequencing was performed in a MiSeq platform (Illumina) using the MiSeq Reagent Kit v3 (2 × 300 base pairs) according to the manufacturer's instructions. Files were recovered (.fastq), and paired-end reads were joined using mothur v.1.35.1 (Schloss et al., 2009). Sequences are available for download via the NCBI short read archive under BioProject PRJNA406945. The following criteria were used to eliminate low-quality reads: average quality (window size = 50) <30, length 460 base pairs, presence of ambiguous characters (“N”), homopolymer <8. The remaining reads were aligned using the SILVA database, trimmed and filtered. Then, sequences were preclustered with diff = 4. Chimeras were detected using UCHIME (Edgar et al., 2011) and excluded. Taxonomic classification was carried out using the RDP database (Release 11) with a confidence threshold of 80%. Sequences not assigned as Bacteria or classified as Chloroplast or Mitochondria were discharged. Singletons and doubletons were removed, and the number of sequences in the 22 samples was normalized to the same number of sequences. Sequences were then used as input to generate a distance matrix and clustered into operational taxonomic units (OTUs) at the sequence similarity cutoff of 97%. Species richness and Shannon diversity index were calculated in mothur. Taxonomic assignment of OTUs was performed using Greengenes (version 13_5).

The OTU relative abundances in the samples were used to generate an ordination plot by nMDS (non-metric multidimensional scaling) based on Bray-Curtis similarity coefficients. The limnological parameters and cyanobacterial cell counts with significant differences between the periods (*t*-test, *p* < 0.001) were plotted together with the nMDS. Statistical significance of sample grouping was tested with PERMANOVA. These analyses were performed on package Past3 (Hammer et al., 2001). To identify the major OTU contributors to grouping differentiation (periods), we used a similarity percentage analysis (SIMPER) (Clarke, 1993). Spearman correlation was used to test the degree of association among limnological variables with cyanobacterial cell counts and cyanobacterial OTUs and to test the association between specific cyanobacterial OTUs and heterotrophic bacterial OTUs (considering only those that contributed at least 0.2% to the total of sequences). We considered relevant correlations those with *p* < 0.001 and *r* > 0.6. The visualization of these correlations was made with the Cytoscape package version 3.4.0 (available at: www.cytoscape.org) using the plugin CoNet. The *r*-value (>0.6) was selected to support the generation of an edge-weighted spring-embedded network (Assenov et al., 2008). The network included the classified OTUs which contributed with at least 0.2% of the total.

## Results

### Limnological characterization

During the study period, Funil Reservoir was characterized by turbid, dynamic, and slightly eutrophic conditions (Table 1). Water transparency varied between 0.25 and 2 m, with lower values between October and early January. Water retention time ranged between 23 and 38 days, with lower values beginning in mid-January, coinciding with the increase in precipitation. The pH was generally alkaline, ranging between 7.1 and 10.9, with highest values on January and February. Nitrogen concentrations were always above the potentially limiting concentration for phytoplankton growth (>100 μg L^−1^), while SRP concentrations were below the detection limit (<2 μg L^−1^) in almost all samples, suggesting strong phosphorus limitation during most of the studied period (Table 1).

**Table 1:** Limnological variables and abiotic parameters associated with the collected samples.

	**Oct-30**		**Nov-27**		**Dec-9**		**Dec-23**		**Jan-9**		**Jan-23**		**Jan-30**		**Feb-20**		**Feb-26**		**Mar-12**		**Mar-26**	
**Points**	**1**	**2**	**1**	**2**	**1**	**2**	**1**	**2**	**1**	**2**	**1**	**2**	**1**	**2**	**1**	**2**	**1**	**2**	**1**	**2**	**1**	**2**
RT	35	35	28	28	33	33	38	38	36	36	31	31	28	28	23	23	24	24	29	29	36	36
Secchi	0.9	0.7	0.7	0.7	0.25	0.8	0.35	0.9	0.4	0.7	0.7	0.8	0.8	0.9	1.1	1.4	1.3	1.6	1.0	1.2	1.4	2.0
Temp	24.5	24.3	24.9	24.9	27.9	27.4	26.9	26.6	29.1	28.8	28.9	28.4	28.7	29.2	28.0	27.9	28.7	28.6	28.5	27.7	27.5	28.9
pH	7.1	8.7	7.5	8.2	8.3	9.4	9.2	8.8	10.5	10.1	10.4	10.4	10.1	10.3	7.9	8.0	9.9	10.9	7.3	8.7	7.2	6.6
DIN	1079	1062	858	695	1038	265	402	437	435	453	307	435	665	597	700	829	171	505	507	485	437	577
SRP	28.7	<2.0	<2.0	<2.0	27.0	<2.0	<2.0	<2.0	12.0	<2.0	<2.0	<2.0	<2.0	<2.0	31.3	<2.0	<2.0	<2.0	<2.0	18.3	33.4	<2.0
TP	44.8	19.0	10.7	8.5	40.5	40.9	25.2	6.3	36.3	10.5	7.4	9.4	6.1	6.8	42.8	16.6	25.6	15.0	3.5	45.9	56.3	26.0
Cyano	1.0	2.0	3.0	3.0	5.0	7.0	6.0	5.0	8.0	4.0	8.0	4.0	3.0	2.0	2.0	1.0	3.0	1.0	1.0	2.0	1.0	2.0
H'	4.5	4.0	4.7	4.5	3.6	5.0	5.0	3.6	4.6	4.3	4.2	4.8	3.3	3.6	4.5	4.0	4.0	4.3	4.6	4.7	3.2	4.9
S	1718	997	1488	1347	613	973	801	702	1222	998	662	1170	1074	1706	2038	1781	1037	960	1241	1314	1835	1664

*1 and 2 corresponds to sampling points 1 (central part of the reservoir) and 2 (dam). RT, retention time (days); Secchi, water transparency (m); Temp, water temperature (°C); DIN, dissolved inorganic nitrogen (μg L^-1^); SRP, soluble reactive phosphorous (μg L^-1^); TP, total phosphorous (μg L^-1^); Cyano, cyanobacterial biomass (mg L^-1^); H', Shannon index; S, Bacterial Richness*.

### Cyanobacterial dynamics

**Figure 1:**
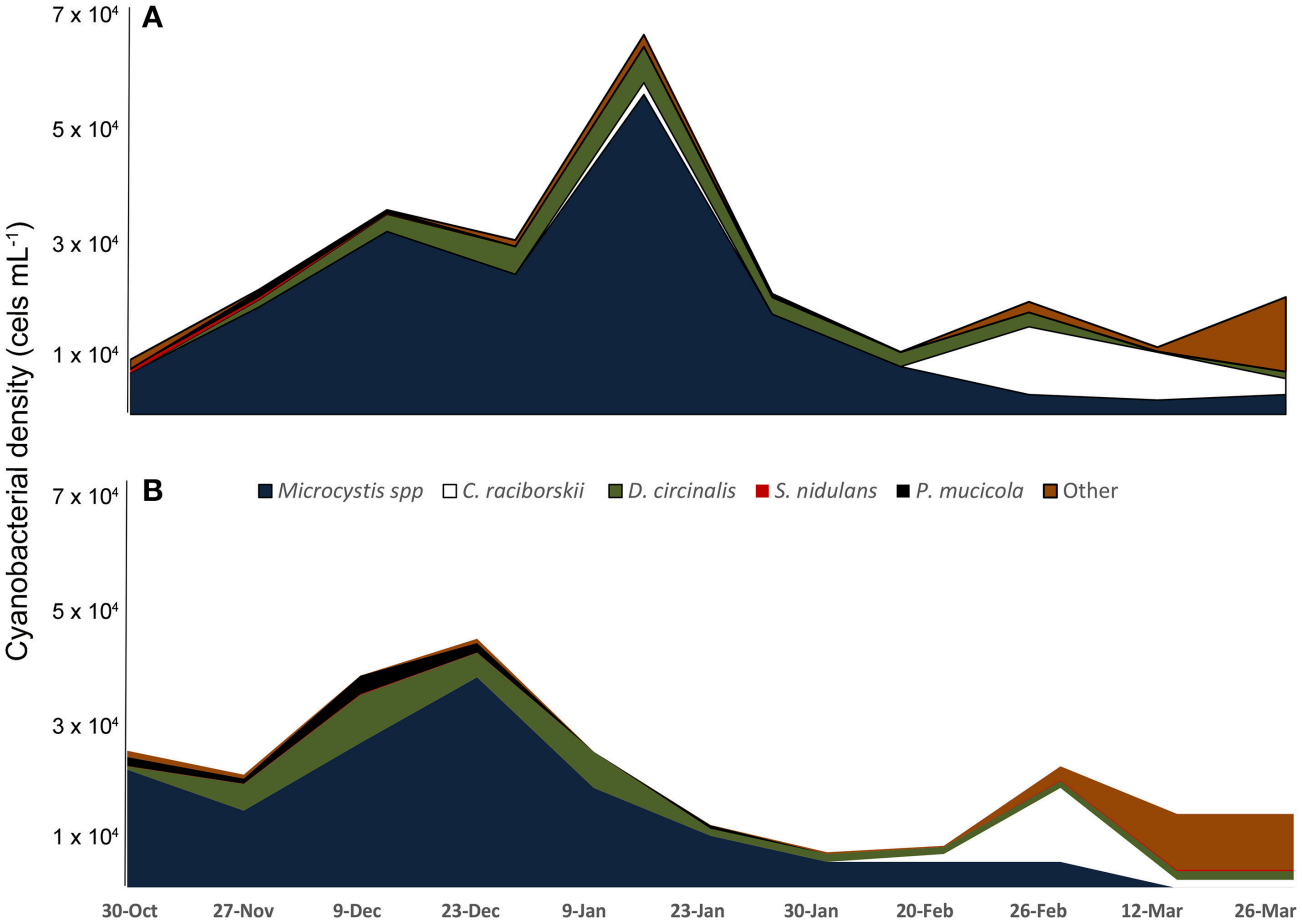
Variation of cyanobacteria density from October 2013 to March 2014 in two locations of Funil reservoir **(A)** point 1, central part, **(B)** point 2, near the dam.

Microscopic analysis revealed that cyanobacteria were the dominant (96.2–99.8%) component of phytoplankton all over the sampling period; 22 cyanobacterial species were identified (Supplementary Table 1). The most abundant species were *Microcystis* spp. (referred herein as *Microcystis aeruginosa* complex, MAC)*, Dolichospermum circinale, Cylindrospermopsis raciborskii, Pseudanabaena mucicola, Synechococcus nidulans*, and *Synechocystis aquatilis* (Figure 1). An MAC bloom was apparent from October to mid-January with the co-occurrence of *D. circinale* and *P. mucicola*. Beginning in mid-January, *C. raciborskii* dominated the phytoplankton community together with *D. circinale, S. nidulans*, MAC and other cyanobacteria. Although the cyanobacterial biomass differed between the two sampling points during the period, the relative contribution of the cyanobacterial species was similar in the two locations (Figure 1).

**Figure 2:**
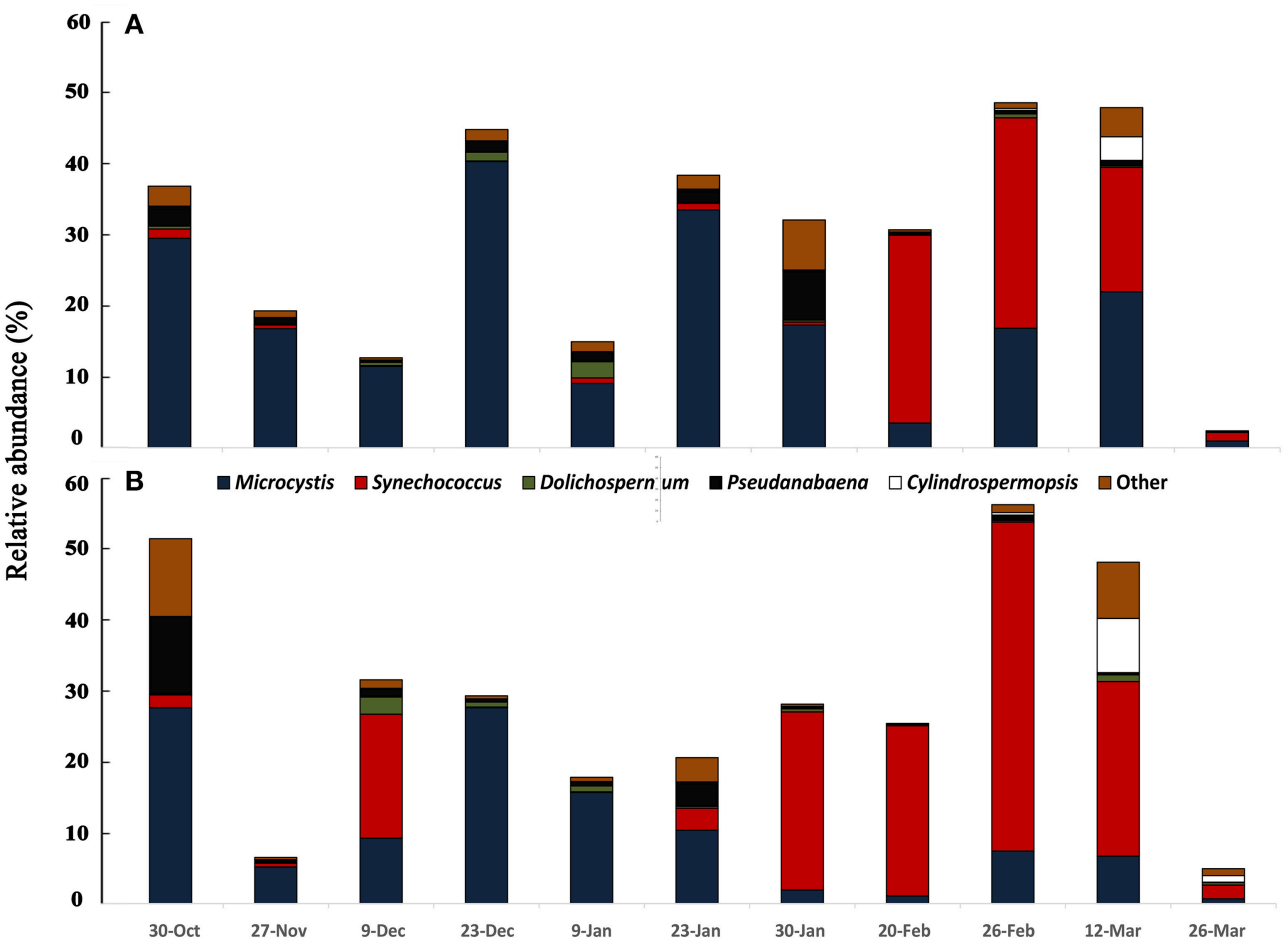
Variation of cyanobacterial community accessed by 16S rDNA sequencing from October 2013 to March 2014 in two sampling points of Funil reservoir **(A)** point 1, central part, **(B)** point 2, near the dam.

The cyanobacterial community composition assessed by *16S rDNA* sequencing revealed a somewhat different pattern for the mixed bloom. The main cyanobacteria genera identified were *Microcystis, Dolichospermum, Cylindrospermopsis, Pseudanabaena*, and *Synechococcus* (Figure 2). *Microcystis* dominated from October to end of January, as also observed with microscopy, but the dominance of *C. raciborskii* was not as evident. Instead, a *Synechococcus* predominance followed the decay of the *Microcystis* bloom. This pattern was evident in the two sampling points despite some differences in specific dates.

### Microbial community structure

The metagenomic sequencing of the bacterioplankton community resulted in a total of 2,547,075 sequences (101,883 per sample) that clustered into 6,452 OTUs (3% dissimilarity). Rarefaction curves showed an excellent coverage (Supplementary Figure 2) for all samples. The bacterial richness varied from 613 to 2,038 OTUs, and Shannon indices varied from 3.2 to 5.0 (Table 1).

**Figure 3:**
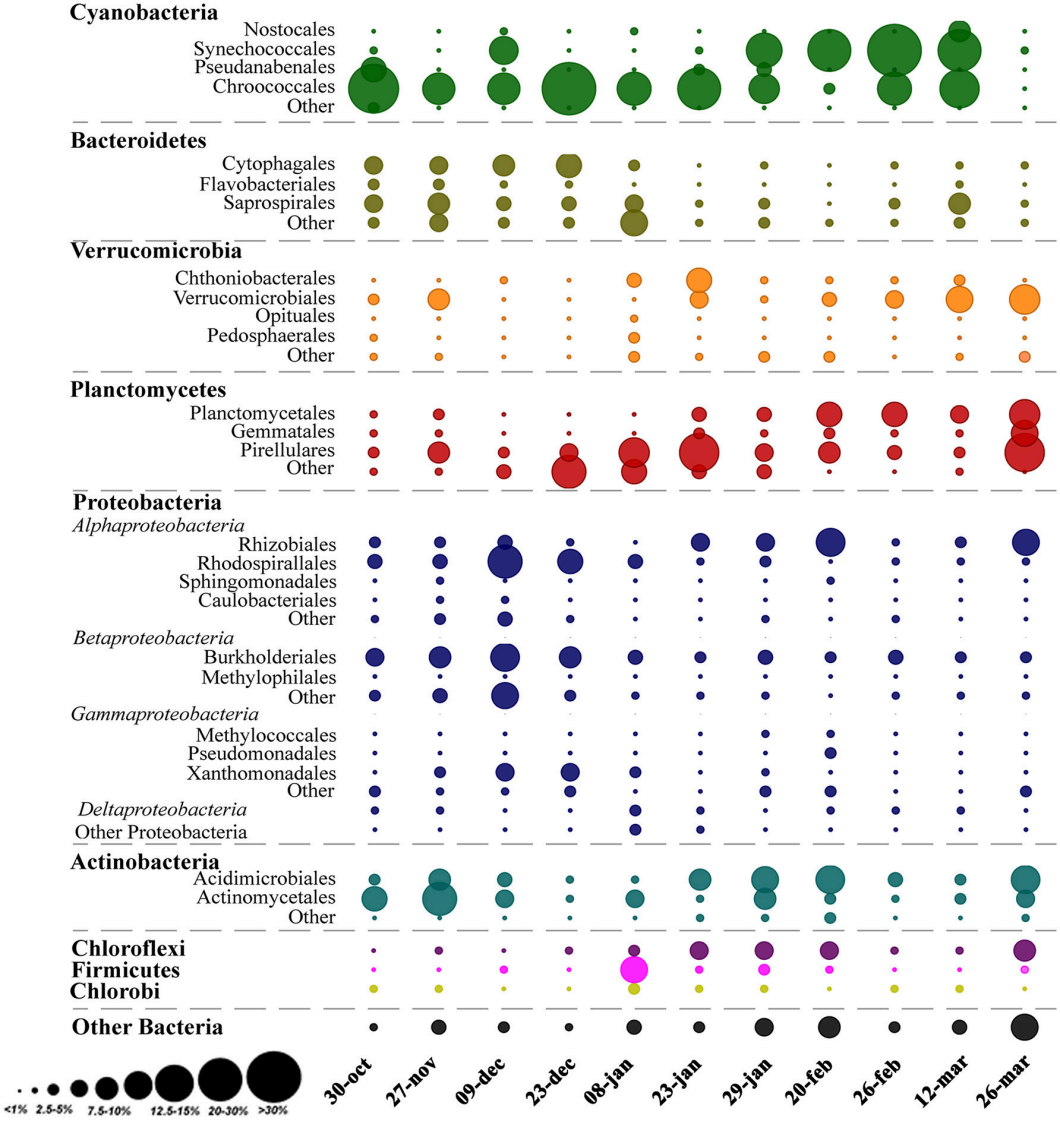
Relative abundance of OTUs classified as Order and Phyla across the sampling period. The area of the bubbles represents the relative abundance of OTUs (average values of the two sampling points). The color of the bubbles indicates the Phylum to which the OTUs were assigned.

**Figure 4:**
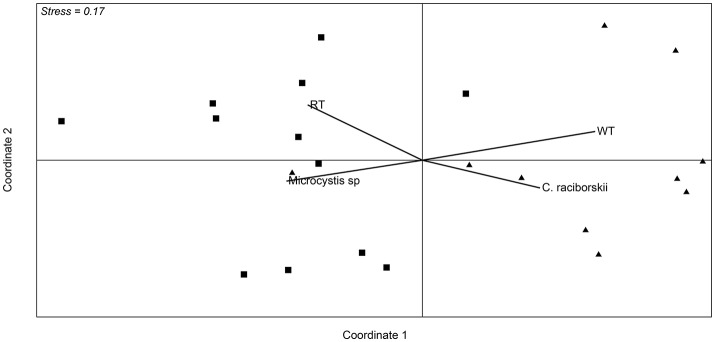
Non-metric multidimensional scaling ordination based on Bray-Curtis similarity of data from OTU abundance in the samples from the two sampling points. Squares correspond to samples from October 2013 to mid January 2014, and triangles correspond to samples from the end of January 2014 to March. Vectors are environmental variables and cyanobacterial microscopic counts that were significantly different between the two defined periods (*p* < 0.01). RT (retention time) WT (water transparency).

Analysis of the whole microbial community showed that Cyanobacteria and Proteobacteria were the dominant phyla followed by Actinobacteria, Bacteroidetes, Verrucomicrobia, and Planctomycetes (Figure 3). The relationship between all samples was assessed using an NMDS based on the bacterial community distribution at OTUs level (3% dissimilarity). The ordination showed clustering of the samples according to the two periods. The difference between these periods was supported by PERMANOVA analysis (*F* = 3.19, *p* = 0.0001). Period 1 corresponded to samples from October to mid-January (*Microcystis* dominance), and period 2 from mid-January to March (*Synechococcus* dominance; Figure 4). For spatial variability, there was no significant difference between the sampling points (*F* = 1.13, *P* = 0.29). The distinction between these two periods was also supported by some limnological parameters and cyanobacterial microscopic counts. Water retention time and transparency showed significant differences between periods one and two [*t*_(20)_ = 2.8, *p* < 0.01 and *t*_(20)_ = −4.9, *p* < 0.01, respectively]. *Microcystis* spp. and *C. raciborskii* cell counts were significantly different between periods [*t*_(16)_ = 3.2, *p* < 0.01 and *t*_(16)_ = −3.29, *p* < 0.01, respectively; Figure 4].

**Figure 5:**
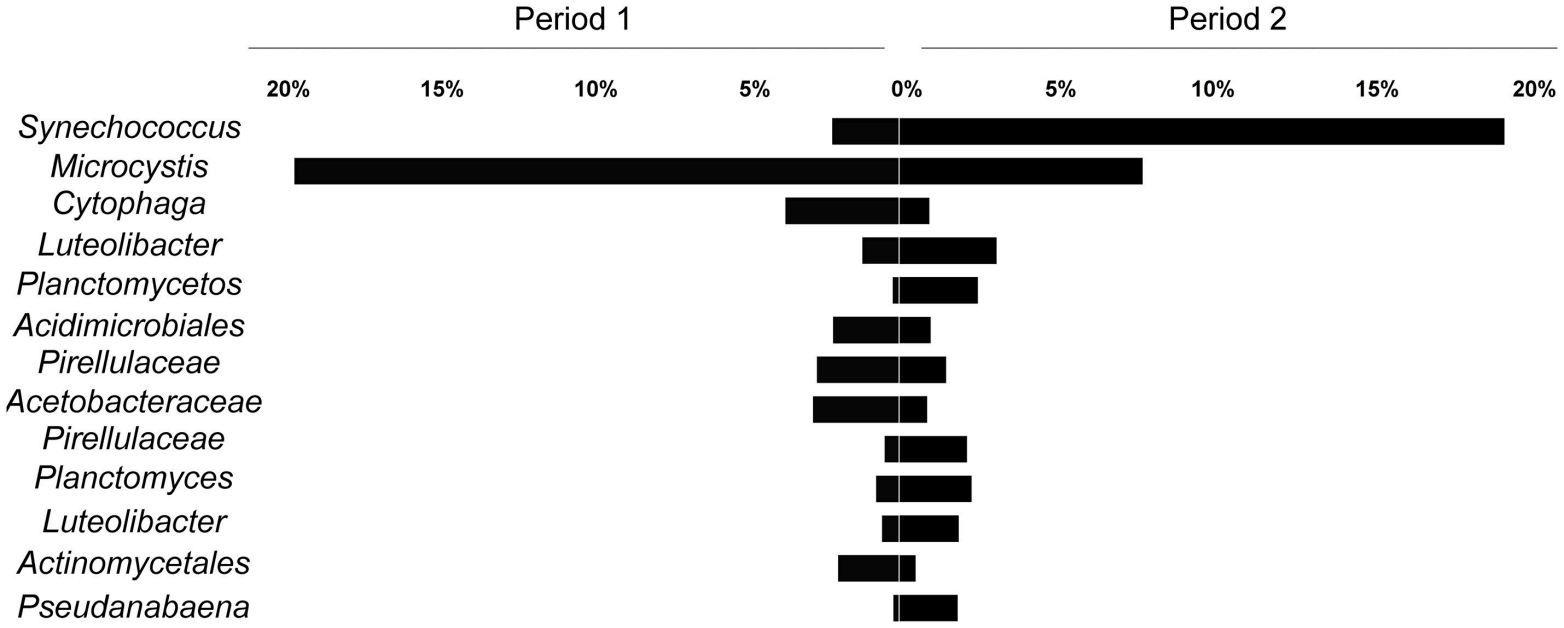
Average relative contribution of OTUs in the two defined periods (Period 1 from October to mid January and Period 2 from mid January to March). The selected OTUs contributed to at least 2% for the differentiation between the periods (SIMPER analysis).

Regarding the main contributors to the distinction between the two periods, SIMPER analysis revealed that OTUs assigned as *Synechococcus* and *Microcystis* accounted for 12.75 and 10.13% of the variability, respectively. Considering other OTUs with over 1% relative abundance, 11 orders also contributed to the variability of the two periods, although their combined impact was <2% (Figure 5).

### Associations of biotic and abiotic factors during the bloom

The data pointed to a clear distinction of a *Microcystis* dominated bloom followed by a *Synechococcus* dominated period. The *16S rDNA* sequencing and microscopy data revealed the correlations among the cyanobacterial taxa. *Microcystis* was positively correlated with *Dolichospermum* (both for microscopy counts and *16SrDNA* data). *C. raciborskii* microscopy counts were positively correlated with *Synechococcus* (*16S rDNA*), while *Pseudanabaena*, which was a contributor to the transition between periods 1 and 2, was negatively correlated with *Microcystis* and positively with *C. raciborskii* (Table 2).

**Table 2:** Values of Spearman correlations (r) among cyanobacterial taxa considering both 16S rDNA sequencing (OTU) and microscopy data and limnological parameters (^*^p < 0.05, ^**^p < 0.001).

	**Temp**	**RT**	**DIN**	**Secchi**	***Microcystis* spp**.	***C. raciborskii***	***D. circinalis***	**SYN OTU**	**MIC OTU**	**DLC OTU**	**PSD OTU**
RT	−0.31										
DIN	−0.44	−0.04									
Secchi	0.14	−0.31	−0.05								
*Microcystis* spp.	−0.17	0.74^**^	−0.18	−0.74^**^							
*C. raciborskii*	0.49	−0.33	−0.22	−0.58^*^	−0.49^*^						
*D. circinale*	−0.07	0.47	−0.62^*^	−0.51^*^	0.63^**^	−0.31					
SYN OTU	0.45^*^	0.62^**^	−0.22	−0.55^**^	−0.65	0.57^**^	−0.57				
MIC OTU	0.09	0.40	0.05	−0.49^*^	0.44	−0.44	−0.06	−0.43			
DLC OTU	0.13	0.25	−0.33	−0.21	0.36^**^	−0.07	0.60^**^	−0.15	0.07		
PSD OTU	0.14	−0.34^*^	0.01	0.74^*^	−0.81^*^	0.44^*^	−0.65	0.71	−0.58^**^	−0.43	
CR OTU	−0.3	−0.04	−0.19	−0.09	0.19	−0.26	0.27	−0.09	−0.17	0.20	−0.41

*RT, retention time (days); Secchi, water transparency (m); Temp, water temperature (°C); DIN, dissolved inorganic nitrogen (ug L-1); SYN, Synechococcus; MIC, Microcystis; DLC, Dolichospermum; PSD, Pseudoanabaena; CR, C. raciborskii. Microcystis spp., C. raciborskii, D. circinale, microscopy count of these species*.

Considering abiotic factors, *Microcystis* microscopic counts (MO) were positively correlated with water retention time and were negatively correlated with water transparency; *C. raciborskii* (MO) was negatively correlated with water transparency; *Dolichospermum* (MO) was negatively correlated with DIN and water transparency; *Synechococcus* (OTU) was positively correlated with temperature and retention time and was negatively correlated with water transparency (Table 2).

**Figure 6:**
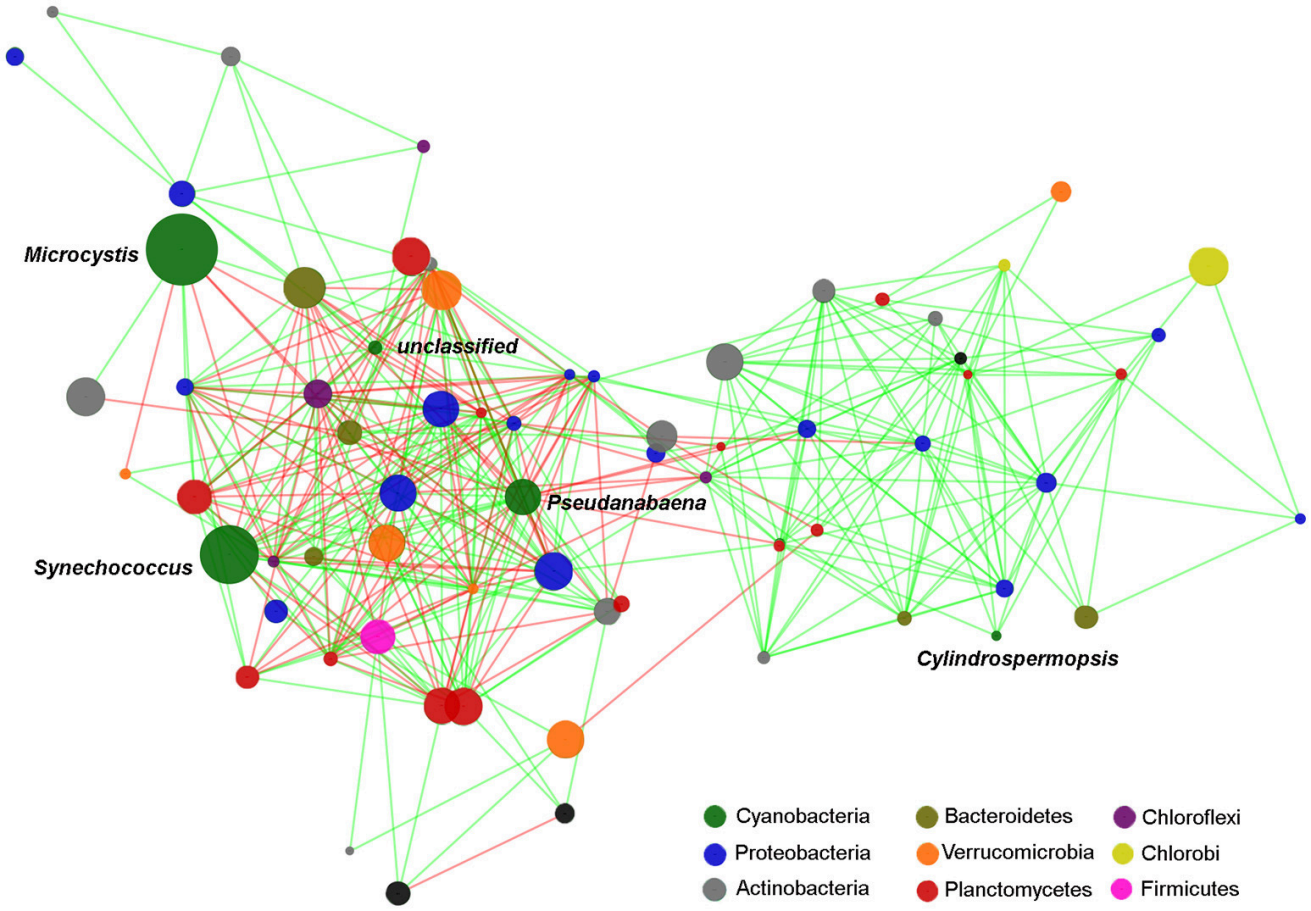
Edge-weighted Spring-embedded network with significant correlations (*p* < 0.001 and *r* > 0.6) between cyanobacterial and heterotrophic bacterial OTUs. Node size is proportional to the OTU abundance and the colors indicate the Phylum to which the OTUs were assigned. Line colors are indicative of the Spearman correlation coefficient (green = positive and red = negative). The network included the OTUs which contributed with at least 0.2% of the total. Centroid sequences of the dominant cyanobacterial OTUs are listed on Supplementary File 1.

Several significant correlations were demonstrated between cyanobacterial taxa and heterotrophic bacteria taxa (Figure 6, Table 3). Regarding the dominant cyanobacteria, a few correlations, mostly negative, were observed between *Microcystis* and other bacterial OTUs, particularly with OTUs from Proteobacteria and Chloroflexi. *Cylindrospermopsis*, the least abundant among the known main Cyanobacteria, showed an opposite pattern with most of the correlations being positive, mainly with OTUs from Proteobacteria and with Planctomycetes. *Synechococcus* showed a higher number of interactions compared to *Microcystis*, and *Cylindrospermopsis* and those were more balanced between positive and negative correlations. *Synechococcus* showed numerous correlations with Proteobacteria, particularly with taxa from the family Comamonadaceae. The *Dolichospermum* OTU showed only one positive correlation with an OTU from Comamonadaceae. *Microcystis* and *Cylindrospermopsis* did not correlate with any other Cyanobacteria, while *Synechococcus* and *Pseudanabaena* showed positive correlation with each other.

**Table 3:** Significant (*p* < 0.001) Spearman correlations (*r* > 0.6) among the main cyanobacterial OTUs and heterotrophic bacteria.

**Cyanobacterial OTU**	**Bacterial OTUs**		***r***
*Microcystis*	Actinobacteria(p)	EB1017 (f)	0.78
		ACK-M1 (f)	−0.66
	Proteobacteria(p)	*Hyphomicrobium* (g)	−0.71
		Betaproteobacteria (c)	−0.69
		Burkholderiales (o)	−0.67
		Burkholderiales (o)	−0.66
		*Limnohabitans* (g)	−0.62
		Sphingomonadales (o)	0.68
	Firmicutes(p)	Bacillales (o)	−0.69
	Chloroflexi(p)		−0.62
		WCHB1-50 (o)	−0.65
		SBR1031 (o)	−0.61
		WCHB1-50 (o)	0.63
	Verrucomicrobia (p)		−0.65
	Planctomycetes(p)	*Planctomyces* (g)	−0.63
	Gemmatimonadetes(p)	Gemmatimonadaceae (f)	0.69
*Dolichospermum*	Proteobacteria(p)	Comamonadaceae(f)	0.61
*Cylindrospermopsis*	Actinobacteria(p)	Actinomycetales (o)	0.82
		ACK-M1 (f)	0.62
		Candidatus_Aquiluna (f)	0.60
	Gemmatimonadetes(p)	KD8-87 (o)	0.81
		*Gemmatimonas* (g)	0.77
	Planctomycetes(p)	Pirellulales (o)	0.81
		Gemmataceae (f)	0.72
		Pirellulaceae (f)	0.65
		CL500-15 (o)	−0.63
	Proteobacteria(p)	*Roseococcus* (g)	0.73
		Betaproteobacteria (c)	0.69
		Acetobacteraceae (f)	0.64
		Vibrionales (o)	0.62
	OD1(p)		0.71
	Bacteroidetes(p)	Chitinophagaceae (f)	0.61
	Chloroflexi(p)	A4b (f)	0.61
*Synechococcus*	Verrucomicrobia(p)	*Prosthecobacter*(g)	0.84
		*Luteolibacter*(g)	0.62
		LD19(f)	−0.66
	Proteobacteria(p)		0.83
		Comamonadaceae(f)	−0.67
		Comamonadaceae(f)	−0.65
		Comamonadaceae(f)	0.64
		Comamonadaceae(f)	0.67
		Comamonadaceae(f)	0.71
		Enterobacteriaceae(f)	0.77
		Oxalobacteraceae(f)	0.61
	Chloroflexi(p)		0.77
	Actinobacteria(p)	C111(f)	0.70
		C111(f)	−0.66
		Actinomycetales(o)	0.60
		Solirubrobacterales(o)	0.69
		*Mycobacterium*(g)	0.64
	Planctomycetes(p)	Pirellulaceae(f)	0.67
		Pirellulaceae(f)	−0.68
		*Planctomyces*(g)	0.62
	Cyanobacteria(p)	*Pseudanabaena*(g)	0.71
	Armatimonadetes(p)	Armatimonadaceae(f)	0.70
	Bacteroidetes(p)	*Fluviicola*(g)	−0.66
	Firmicutes(p)	Bacillales(o)	0.65
	Acidobacteria(p)	iii1-15(o)	0.65
	Bacteroidetes(p)	Chitinophagaceae(f)	−0.63

*Taxonomic assignment according to Greengenes databases. p, Phyla; c, Class; o, Order; f, Family; g, Genus. The analysis included those OTUs that contributed with ate least 0.2% of the total of OTUs per sample*.

## Discussion

In this study, we investigated the bacterial community coupled with a mixed cyanobacterial bloom (initially dominated by *Microcystis* and *Dolichospermum*, followed by *C. raciborskii* and *Synechococcus*) occurring in a tropical reservoir during late spring-summer, combining microscopy with metagenomics. Temporal shifts in the dominance of bloom-forming cyanobacterial genera were associated with physical features of the water and with shifts in the associated heterotrophic bacteria. Changes in abiotic factors during this period probably influenced both the cyanobacterial and the non-cyanobacterial microbial community. To gain insights about possible biotic associations during a cyanobacterial bloom in this tropical system we have explored specific interactions of the main cyanobacterial genera with some components of the heterotrophic bacterial community.

### Cyanobacterial dynamics

For the last several decades the Funil Reservoir has experienced cyanobacterial blooms, mainly during the summer, dominated by *Microcystis, Dolichospermum*, and *Cylindrospermopsis* (Soares et al., 2013; Guedes et al., 2014; Rangel et al., 2016), which are also the major bloom-forming genera globally (O'Neil et al., 2012; Antunes et al., 2015; Harke et al., 2016). The succession of a bloom dominated by *Microcystis* and *Dolichospermum* by the dominance of *C. raciborskii* during the summer has been reported for several years in this system, and it has been more tightly associated with temperature and physical variables than with nutrient availability (Soares et al., 2012). Thus, the stratification of the water column and the reservoir residence time are important abiotic factors associated with the bloom dynamics (Soares et al., 2009; Rangel et al., 2016). In the present study, the *Microcystis* bloom was associated with high retention time and low water transparency. Similarly, previous studies in this reservoir have associated the dominance of *Microcystis* with a more stable and prolonged period of thermal stratification (Soares et al., 2009, 2012; Rangel et al., 2016). *M. aeruginosa* is considered a species adapted to high light intensities (Robarts and Zohary, 1987); it can thus form blooms on the surface. As a consequence, *Microcystis* can outcompete other species by reducing available light for nonbuoyant phytoplankton competitors (Harke et al., 2016). Indeed, in the initial period of the bloom, *Microcystis* accounted for 89% of the total cyanobacterial density. In addition, this species is sensitive to mixing and turbulence (Reynolds, 2006), which can explain the decline of its abundance in the middle of January (Period 1), when precipitation increased and retention time decreased.

After the decay of the *Microcystis* bloom, *C. raciborskii* and *Synechococcus* were the dominant cyanobacteria. Opposite to the former species, the latter two species were positively correlated with lower retention time and higher water transparency. Previous investigations in this reservoir have associated *C. raciborskii* blooms with periods of water column mixing (Soares et al., 2009, 2012). This species is considered to tolerate both stratified and mixed conditions and can dominate in mixed periods (Berger et al., 2006; Bonilla et al., 2012; Soares et al., 2013). This is likely due to the ability of cells to photoadapt to dark and fluctuating light conditions (O'Brien et al., 2009).

The period of *C. raciborskii* dominance was coupled with the high relative abundance of *Synechococcus* (shown by *16S rDNA* sequences). This has never been reported for this reservoir, since previous studies characterized the phytoplankton community by microscopy only. Picoplanktonic cells (size 0.2–2 μm) seem to be generally subquantified by the Utermöhl method, while other techniques such as fluorescence microcopy and flow cell cytometry are more efficient for their quantification (Callieri, 2007). The high relative abundance of *Synechococcus* in conditions of higher water transparency and low retention time is in accordance with the high light requirement reported for this genus (Reynolds et al., 2002), which together with its high growth rate can explain its rise just after the senescence of the *Microcystis* bloom.

### Microbial community dynamics

In the present study, we found a clear distinction between the heterotrophic bacteria associated with the two periods of the cyanobacterial bloom. The distinction of these periods was based on limnological parameters (water retention time and transparency) and cyanobacterial community composition (*Microcystis* spp. and *C*. *raciborskii/Synechococcus* dominance). While the influence of the changes in abiotic environmental parameters on the composition of the bacterioplankton community cannot be disregarded, we discuss the overall community composition shifts emphasizing the links between the dominant cyanobacterial genera and the associated bacterioplankton in this reservoir.

Changes in the composition of the bacterioplankton community can be linked to phytoplankton blooms (Xing et al., 2007; Li et al., 2015; Woodhouse et al., 2016). Recent studies noted specific associations between heterotrophic bacteria taxa and cyanobacteria, which has been attributed to different dissolved organic matter qualities and quantities produced by different cyanobacteria (Bagatini et al., 2014; Louati et al., 2015). In our case, we did not observe a correlation between cyanobacterial abundance and diversity of the microbial community.

In this study, the major observed bacteria phyla were Cyanobacteria and Proteobacteria followed by Actinobacteria, Bacteroidetes, Verrucomicrobia, and Planctomycetes (Figure 3). Actinobacteria is one of the most frequent phyla in freshwater environments and is reported as highly abundant in different lakes from oligotrophic to eutrophic systems (Newton et al., 2011). This phylum has not been found in physical association with Cyanobacteria but can be a major part of the bacterioplankton community during phytoplankton blooms (Kormas et al., 2010; Newton et al., 2011; Steffen et al., 2017). On the other hand, Bacteroidetes, Verrucomicrobia, and Planctomycetes frequently reach high relative abundance during cyanobacterial blooms (Li et al., 2015; Woodhouse et al., 2016; Parulekar et al., 2017; Steffen et al., 2017). In this study, Verrucomicrobia ranged from 1 to 18% of relative abundance. Bacteria from this phylum are described as able to degrade algal polysaccharides and organic matter, and so can be favored by high cyanobacterial abundances (Bagatini et al., 2014; Woodhouse et al., 2016; Parulekar et al., 2017). The relative abundance of Bacteroidetes also increased during the bloom, and other studies have shown that some taxa in this phylum, such as *Sphingobacteria* and *Flavobacteria*, are often found in high abundance after phytoplankton bloom decay either adjacent or attached to phytoplankton (Li et al., 2011; Bagatini et al., 2014). In the present study, the most abundant Bacteroidetes taxa were Cytophagales and Saprospirales, and higher abundances were associated with the *Microcystis* bloom. Planctomycetes can be abundant in nutrient-enriched waters (Woebken et al., 2007), which frequently have a high abundance of phytoplankton. Other studies also reported higher densities of Planctomycetes after diatom (Morris et al., 2006) or cyanobacterial blooms (Eiler and Bertilsson, 2004), suggesting a possible association of this phylum with phytoplankton. For example, it has already been observed that Planctomycetes prefer to remain attached rather than be free-living (Allgaier and Grossart, 2006). The functional roles of these associations between cyanobacteria and heterotrophic bacterial taxa are still unclear but likely will be revealed from studies exploring the microbial community functional response during a bloom and the links to environmental conditions (Steffen et al., 2012, 2017).

Previous studies that evaluated an entire year in this reservoir defined the cyanobacteria bloom period as October–March (Soares et al., 2009; Guedes et al., 2014; Rangel et al., 2016). Thus, in the period evaluated here, cyanobacterial cell density was always high, so we could not distinguish between situations with and without a bloom. The temporal analysis of the variation in the composition of the bacterial community was related to the abundance of the main cyanobacterial genera that dominated the phytoplankton community. In this late spring-summer bloom, two periods could be distinguished; the first period was characterized by higher retention time, low transparency and dominance of *Microcystis*. This period was also characterized by the high abundance of Bacteroidetes OTUs, particularly Cytophagales. Some bacteria of the genus *Cytophaga* are described as predatory agents in freshwater and marine systems (Daft and Stewart, 1971; Imai et al., 1993; Rashidan and Bird, 2001; Kirchman, 2002). In a previous study, Daft and Stewart (1971) first described a *Cytophaga* strain whose dynamics closely followed *Microcystis* dynamics; the species was able to terminate a bloom. Rashidan and Bird (2001) found a close relationship between the abundance of *Anabaena* sp. and a lytic strain of *Cytophaga*; they suggested that one reason for the decay of the *Anabaena* bloom was the lysis induced by this *Cytophaga* strain. Moreover, in a broad study that followed the microbial community of Lake Champlain by for 8 years using *16S rDNA* sequencing, Tromas et al. (2017) defined *Cytophaga* as a bloom biomarker. In the present study, Cytophagales OTUs co-occurred with *Microcystis* in the first period of the bloom. Thus, we can speculate that the lytic capacity of these bacteria affects other cyanobacteria but not *Microcystis*, that the lytic capacity differentially affects various strains of *Microcystis*, or that such predatory activity is present but is not sufficient to decrease this population of cyanobacteria. Altogether, these observations indicate that *Cytophaga* can be an important biotic factor contributing to the prevalence of the *Microcystis* bloom in the Funil Reservoir.

Among the numerous negative correlations observed for *Microcystis* were those with the genera *Planctomyces, Limnohabitans*, and *Hyphomicrobium*. These genera have already been associated with *Microcystis* by others, but establishing positive correlations. Cai et al. (2013) observed an increased abundance of Planctomycetes associated with *Microcystis* colonies and pointed to a possible role of Planctomycetes in the degradation of sulfated polysaccharides produced by cyanobacteria. Comparing the particle-associated and free-living bacteria community from a lake during a *M. aeruginosa* bloom, Yang et al. reported an increase in the relative abundance of *Limnohabitans* and *Limnobacter*, genera known for utilizing algal-derived dissolved organic matter (Yang et al., 2017). Finally, *Hyphomicrobium* has also been identified in bacterial communities associated to *M. aeruginosa*, involved in cyanopeptide degradation (Briand et al., 2016; Tsao et al., 2017). In the present study, rare positive correlations were found between *Microcystis* and heterotrophic bacteria, among them with the order Sphingomonadales which includes strains from *Sphingopyxis* and *Sphingomonas* that can degrade microcystin (Kormas and Lymperopoulou, 2013).

The second period of the summer bloom was characterized by the dominance of *Synechococcus* (*16S rDNA*) and *C. raciborskii* (MO), which coincided with lower retention time and less turbidity. In this period, Planctomycetes increased in relative abundance, and five Planctomycetes OTUs were positively correlated with *Synechococcus* or *C. raciborskii*. Specifically, we observed a strong correlation between *Planctomyces* and *Synechococcus*. Ruber et al. (2017) also observed high Planctomycetes relative abundance (up to 25%) in a lake dominated by *Synechococcus*. *Planctomyces* was also identified as the most abundant genus within the phylum Planctomycetes in a bacterioplankton community of a deep alpine lake in which *Synechococcus* accounted for circa 30% of cyanobacteria (Salmaso et al., 2017). We also identified positive correlations between *Synechococcus* and other genera such as *Prosthecobacter, Luteolibacter, Mycobacterium, Pseudanabaena*, and *Fluviicola*. The co-occurrence of *Synechococcus* with all these taxa has been reported in natural communities (Bertos-Fortis et al., 2016; Salmaso et al., 2017) and members of *Flavobacteriales* have been identified as dominant associated heterotrophic bacteria in cultures of *Synechococcus* isolates, (Zheng et al., 2017).

In contrast to what has been reported for *Microcystis* and *Synechococcus*, little is known about bacterial groups directly associated with *C. raciborskii*. Here, we observed positive correlations between *C. raciborskii* and the genera *Gemmatimonas* and *Roseococcus*. Gemmatimonas related OTUs have been identified in bacterial communities associated with *C. raciborskii* cultures (Bagatini et al., 2014).

It is important to highlight that any disagreement observed in the correlation dynamics from the present study to others may represent real differences in biological dynamics, but also can be derived from the use of different methodologies. The present study has the advantage of presenting a very comprehensive survey of the microbial community in the reservoir, but as many studies using the next generation sequencing, it lacks reliable taxonomical resolution at the species level, which limits the description of ecologically relevant correlations with reliability.

While other studies have analyzed the bacterial community associated with blooms of cyanobacteria by considering factors associated with the rise and decay of blooms, some investigations, including the present study, focused on variations that occur during a bloom period; this allowed us to detail not only the influence of cyanobacteria biomass but also the possible favoring of different bacterial communities by different cyanobacterial species (or *vice versa*). This approach is a first step to understanding the mechanisms associated with the shift of the dominance of cyanobacteria genera, which until now have been based almost solely on interactions that involve cyanobacteria alone such as direct competition or allelopathy. Future studies under laboratory conditions can simulate the interactions among the cyanobacterial and bacterial genera described here, evaluating possible synergistic or antagonist relationships, with the potential to develop biocontrol tools for cyanobacterial blooms.

## Conclusion

Our study has provided insights on the bacterial communities associated with bloom-forming cyanobacteria in a tropical system. Temporal shifts in the dominance of cyanobacteria genera were associated not only with physical features of the water (retention time and transparency) but also with shifts in the associated heterotrophic bacteria. Our results suggest specific interactions of the main cyanobacterial genera with certain groups of the heterotrophic bacterial community, but further studies exploring the microbial community functional aspects and environmental conditions are needed to better understand the ecophysiological role of these associations.

## Author contributions

IG: Conceived the study design, conducted the sampling, performed sequencing and data analysis and wrote the paper; CR: Conducted the bioinformatic analysis; LR: Conducted sampling, phytoplankton quantification, and analyzed data; LS: Analyzed data and edited the manuscript; PB: Provided counseling in sequencing and edited the manuscript; SA: Conceived the study design, analyzed data and edited the manuscript; AP: Conceived the study design, performed sequencing and data analysis, and wrote the paper. All authors approved the final submitted manuscript.

### Conflict of interest statement

The authors declare that the research was conducted in the absence of any commercial or financial relationships that could be construed as a potential conflict of interest.

## Supplementary material

Supplementary Figure 1 Map of the Funil Reservoir showing geographical location and sampling stations (Modified from Soares et al., 2009).

Supplementary Figure 2 Rarefaction curves of 16SrDNA sequences from all samples.

Supplementary Table 1 Cyanobacterial species identified by microscopic analysis.

Supplementary File 1 Dominant cyanobacterial OTUs as represented in Figure 6. OTU identification, number of retrieved sequences for each OTU, genus classification and representative sequence centroid.
