# Supplementary figures and images for: Close Link Between Harmful Cyanobacterial Dominance and Associated Bacterioplankton in a Tropical Eutrophic Reservoir
Source: Front Microbiol. 2018 Mar 12;9:424. doi: 10.3389/fmicb.2018.00424 (PMC5857610; doi:10.3389/fmicb.2018.00424)

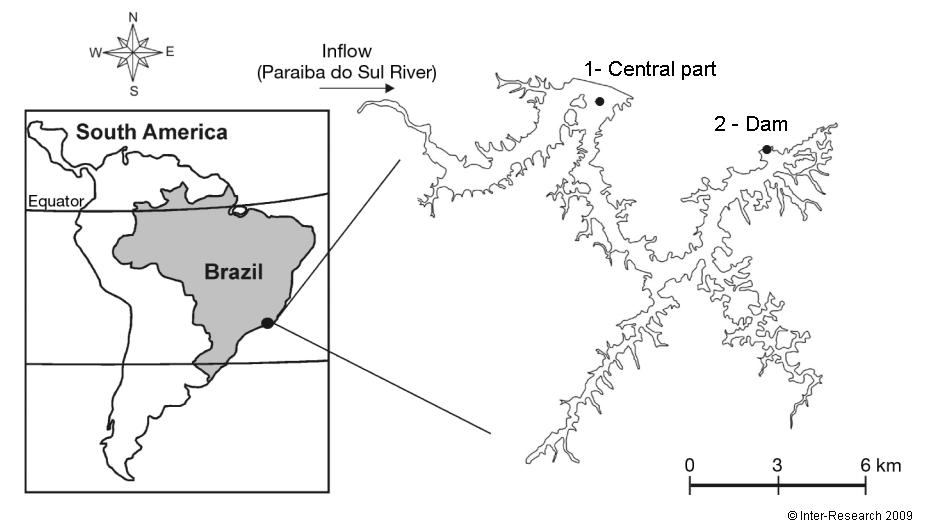

Supplement: Supplementary Figure 1 — Map of the Funil Reservoir showing geographical location and sampling stations (Modified from Soares et al., 2009). [file Image1.tif]

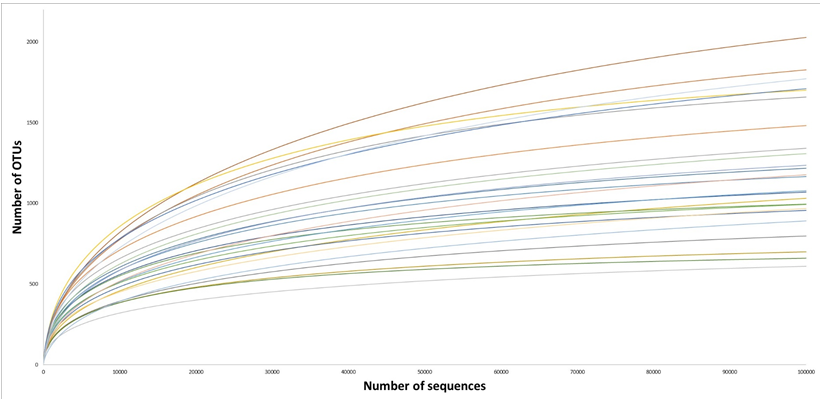

Supplement: Supplementary Figure 2 — Rarefaction curves of 16SrDNA sequences from all samples. [file Image2.TIF]
