# Supplementary material for: Close Link Between Harmful Cyanobacterial Dominance and Associated Bacterioplankton in a Tropical Eutrophic Reservoir
Source: Front Microbiol. 2018 Mar 12;9:424. doi: 10.3389/fmicb.2018.00424 (PMC5857610; doi:10.3389/fmicb.2018.00424)
Supplement: Supplementary Table 1 — Cyanobacterial species identified by microscopic analysis. [file Table1.DOCX]

Supplementary Table 1- Cyanobacterial species identified by microscopic analysis

| *Aphanocapsa delicatissima* |
| --- |
| *Aphanocapsa elachista* |
| *Aphanocapsa incerta* |
| *Aphanothece sp.* |
| *cf Coelomorum tropicale* |
| *cf Coelosphaerium evidenter-marginatum* |
| *Cyanodiction imperfectum* |
| *Cyanogranis ferruginea* |
| *Cylindrospermopsis raciborskii* |
| *Dolichospermum circinale* |
| *Merismopedia tenuissima* |
| *Microcystis aeruginosa* |
| *Microcystis panniformis* |
| *Sphaerocavum brasiliense* |
| *Planktolyngbya limnetica* |
| *Planktothrix isothrix* |
| *Pseudanabaena mucicola* |
| *Radiocystis fernandoi* |
| *Snowella lacustris* |
| *Sphaerocavum brasiliense* |
| *Synechococcus nidulans* |
| *Synechocystis aquatilis* |
