# Supplementary material for: Close Link Between Harmful Cyanobacterial Dominance and Associated Bacterioplankton in a Tropical Eutrophic Reservoir
Source: Front Microbiol. 2018 Mar 12;9:424. doi: 10.3389/fmicb.2018.00424 (PMC5857610; doi:10.3389/fmicb.2018.00424)
Supplement: Supplementary File 1 — Dominant cyanobacterial OTUs as represented in Figure 6. OTU identification, number of retrieved sequences for each OTU, genus classification and representative sequence centroid. [file Table2.DOCX]

Supplementary file 1

Dominant cyanobacterial OTUs as represented in Figure 6. OTU identification, number of retrieved sequences for each OTU, genus classification and representative sequence centroid.

>Otu000001|267017|Synechococcus

CCTACGGGCGGCAGCAGTGGGGAATTTTCCGCAATGGGCGAAAGCCTGACGGAGCAACGCCGCGTGAGGGATGAAGGCCTCTGGGCTGTAAACCTCTTTTCTCAAGGAAGAAGATCTGACGGTACTTGAGGAATAAGCCACGGCTAATTCCGTGCCAGCAGCCGCGGTAATACGGGAGTGGCAAGCGTTATCCGGAATTATTGGGCGTAAAGCGTCCGCAGGCGGTCTTGTAAGTCTGTCGTTAAAGCGTGGAGCTTAACTCCATTTCAGCGATGGAAACTGTAAGACTAGAGTGTGGTAGGGGCAGAGGGAATTCCCGGTGTAGCGGTGAAATGCGTAGATATCGGGAAGAACACCAGTGGCGAAGGCGCTCTGCTGGGCCATAACTGACGCTCATGGACGAAAGCCAGGGGAGCGAAAGGGATTAGATACCCCAGTAGTC

>Otu000002|409415|Microcystis

CCTACGGGAGGCAGCAGTGGGGAATTTTCCGCAATGGGCGAAAGCCTGACGGAGCAACGCCGCGTGAGGGAGGAAGGTCTTTGGATTGTAAACCTCTTTTCTCAAGGAAGAAGTTCTGACGGTACTTGAGGAATCAGCCTCGGCTAACTCCGTGCCAGCAGCCGCGGTAATACGGGGGAGGCAAGCGTTATCCGGAATTATTGGGCGTAAAGCGTCCGCAGGTGGTCAGCCAAGTCTGCCGTCAAATCAGGTTGCTTAACGACCTAAAGGCGGTGGAAACTGGCAGACTAGAGAGCAGTAGGGGTAGCAGGAATTCCCAGTGTAGCGGTGAAATGCGTAGAGATTGGGAAGAACATCGGTGGCGAAAGCGTGCTACTGGGCTGTATCTGACACTCAGGGACGAAAGCTAGGGGAGCGAAAGGGATTAGATACCCTAGTAGTC

>Otu000021|14769|Dolichospermum

CCTACGGGTGGCAGCAGTGGGGAATTTTCCGCAATGGGCGAAAGCCTGACGGAGCAATACCGCGTGAGGGAGGAAGGCTCTTGGGTTGTAAACCTCTTTTCTCAGGGAAGAAGAAAAGACGGTACCTGAGGAATAAGCATCGGCTAACTCCGTGCCAGCAGCCGCGGTAATACGGAGGATGCAAGCGTTATCCGGAATGATTGGGCGTAAAGGGTCCGCAGGTGGCATTGAAAGTCTGCTGTTAAAGAGTTTGGCTCAACCAAATAAGAGCAGTGGAAACTACAAAGCTAGAGTTTGGTCGGGGCAGAGGGAATTCCTGGTGTAGCGGTGAAATGCGTAGATATCAGGAAGAACACCAGTGGCGAAGGCGCTCTGCTAGGCCGAGACTGACACTGAGGGACGAAAGCTAGGGGAGCGAATGGGATTAGATACCCCAGTAGTC

>Otu000026|41675|Pseudanabaena

CCTACGGGGGGCAGCAGTGGGGAATTTTCCGCAATGGGCGAAAGCCTGACGGAGCAATACCGCGTGAGGGAAGAAGGTCTGTGGATTGTAAACCTCTTTTGTTAGGGAAGATAATGACGGTACCTAACGAATAAGCATCGGCTAACTCCGTGCCAGCAGCCGCGGTAAGACGGAGGATGCAAGCGTTATCCGGAATTATTGGGCGTAAAGCGTACGTAGGCGGTTTTATAAGTCTGTTGTCAAAGCCCGAGGCTTAACCTTGGAAAGGCAATGGAAACTGTAAGACTAGAGAGAGATAGGGGCAGGAGGAATTCCAGGTGTAGCGGTGAAATGCGTAGATATCTGGAAGAACACCAGTGGCGAAAGCGTCCTGCTGGATCTCAACTGACGCTGAAGTACGAAAGCTAGGGGAGCGAATGGGATTAGATACCCCTGTAGTC

>Otu000066|13460|Cylindrospermopsis

CCTACGGGAGGCAGCAGTGGGGAATTTTCCGCAATGGGCGAAAGCCTGACGGAGCAATACCGCGTGAGGGAGGAAGGCTCTTGGGTCGTAAACCTCTTTTCTCAAGGAAGAAGAAAGTGACGGTACTTGAGGAATAAGCATCGGCTAACTCCGTGCCAGCAGCCGCGGTAATACGGAGGATGCAAGCGTTATCCGGAATGATTGGGCGTAAAGGGTCTGCAGGTGGAACTGAAAGTCTGCTGTTAAAGAGTTTGGCTTAACCAAATAAAAGCGGTGGAAACTACAGAACTAGAGTGCGGTAGGGGCAAAAGGAATTCCTGGTGTAGCGGTGAAATGCGTAGATATCAGGAAGAACACCGGTGGCGAAAGCGTTTTGCTAGACCGTAACTGACACTGAGGGACGAAAGCTAGGGGAGCGAATGGGATTAGATACCCCAGTAGTC

>Otu000063|22643|Unclassified

CCTACGGGGGGCAGCAGTGGGGAATTTTCCGCAATGGGCGAAAGCCTGACGGAGCAATACCGCGTGAGGGACGAAGGCCTGTGGGTTGTAAACCTCTTTTCTCAGGGAAGAAGATCTGACGGTACCTGAGGAATCAGCATCGGCTAACTCCGTGCCAGCAGCCGCGGTAAGACGGAGGATGCAAGCGTTATCCGGAATTATTGGGCGTAAAGCGTCCGCAGGCGGTTTCGTAAGTCTGTCTTTAAAGAGTGGAGCTTAACTCCATAAAGGGGATGGAAACTGCGAGACTAGAGGTAGGTAGGGGTAGAAGGAATTCCCAGTGTAGCGGTGAAATGCGTAGATATTGGGAAGAACACCAGCAGCGAAGGCGTTCTACTGGACCAAACCTGACGCTCATGGACGAAAGCTAGGGGAGCGAAAGGGATTAGATACCCCAGTAGTC

>Otu000126|2996|Unclassified

CCTACGGGGGGCAGCAGTGGGGAATTTTCCGCAATGGGCGAAAGCCTGACGGAGCAATACCGCGTGAGGGACGAAGGCCTGTGGGTTGTAAACCTCTTTTCTCAGGGAAGAAGCTCTGACGGTACCTGAGGAATCAGCATCGGCTAACTCCGTGCCAGCAGCCGCGGTAAGACGGAGGATGCAAGCGTTATCCGGAATTATTGGGCGTAAAGCGTCCGCAGGCGGTTTCGTAAGTCTGTCTTTAAAGAGTGGAGCTTAACTCCATAAAGGGGATGGAAACTGCGAGACTAGAGGTAGGTAGGGGTAGAAGGAATTCCCAGTGTAGCGGTGAAATGCGTAGATATTGGGAAGAACACCAGCAGCGAAGGCGTTCTACTGGACCAAACCTGACGCTCATGGACGAAAGCTAGGGGAGCGAAAGGGATTAGATACCCCAGTAGTC
